# No Difference in Transverse Abdominis Activation Ratio between Healthy and Asymptomatic Low Back Pain Patients during Therapeutic Exercise

**DOI:** 10.1155/2010/459738

**Published:** 2010-08-31

**Authors:** Nathaniel Gorbet, Noelle M. Selkow, Joseph M. Hart, Susan Saliba

**Affiliations:** Department of Kinesiology, University of Virginia, 210 Emmet Street South, Charlottesville, VA 22904, USA

## Abstract

Dysfunction of the transverse abdominis (TrA) has been associated with LBP. Several therapeutic exercises are prescribed to help target the TrA. Rehabilitative ultrasound imaging (RUSI) is used to capture activation of the TrA during exercise. The purpose was to examine TrA activation during the ADIM and quadruped exercises between healthy and nonsymptomatic LBP patients. We instructed the subjects how to perform the exercises and measured muscle thickness of the TrA at rest and during the exercises using RUSI. This allowed us to calculate TrA activation ratio during these exercises. We found no significant differences between activation ratios of the two groups during either exercise; however TrA activation during the ADIM was higher than the quadruped exercise. These exercises were capable of activating the TrA, which may be in part due to the verbal instruction they received. These exercises could be used during prevention or rehabilitation programs, since the TrA is activated.

## 1. Introduction

Low-back pain (LBP) is a condition that affects about two thirds of the general population [1]. In 90% of patients, an episode of LBP typically subsides within 2 to 4 weeks [2–4]. However, the recurrence rate of LBP is alarmingly high at an estimated rate of 60–80% [5, 6]. A rising focus in research and rehabilitation is on treatment classification of nonspecific LBP into smaller subgroups (manual therapy, specific exercises, traction, and stabilization) so that more specific rehabilitative techniques can be prescribed [7, 8]. The stabilization classification group is often prescribed therapeutic exercises thought to activate the patient's local stabilizers of the back. These local stabilizers have been shown to be atrophied or dysfunctional in patients correctly categorized to the stabilization group [9, 10]. The musculature of the lower back is composed of global movers and local stabilizers, where the global movers are responsible for gross movement and the local stabilizers are thought to stabilize the vertebral segments [11]. The transversus abdominis (TrA) is one of the dynamic stabilizers. In a healthy population, the TrA has been found to be preactivated during upper-extremity movements, but those suffering from LBP have delayed activation patterns [12]. Even after an episode of low-back pain has resolved, the stabilizing muscles do not return to normal function [13]. During an isomeric knee extension/flexion task, LBP patients had varying levels of activation depending on what intervention they had (motor control exercise, general exercise, or spinal mobilization) [14]. However, no control group was assessed. Since the local stabilizing muscles are often deep to other muscles, they can be difficult to evaluate and palpate. The abdominal drawing-in maneuver (ADIM) and quadruped exercises are commonly prescribed to target the TrA during rehabilitation; however, it is unclear how well the TrA is activated during these exercises in patients with low-back pain and whether the exercise can be taught to be performed correctly. 

The ADIM is a fundamental procedure used in many core stability exercises. Both the ADIM and quadruped exercises are two of the more commonly prescribed exercises in the biomechanical and EMG literature used to activate the deep abdominal musculature [15–19]. Because of the difficulty in clinically monitoring the TrA during exercise and the potential for dysfunction in LBP patients, it is important to evaluate the function of the TrA during common therapeutic interventions. Rehabilitative ultrasound imaging (RUSI) permits visualization of the musculature and has been used to examine the function of the TrA [15, 19]. RUSI is a valid tool at measuring muscle activation compared to MRI during an abdominal hollowing task with a correlation of  .87 [20]. However, this relationship may change depending on the exercise performed and the intensity of the exercise [21]. The TrA is also difficult to contract properly, and this tool allows for immediate feedback on performance. Comparing the activation of the TrA in LBP subjects to that of healthy controls can help discern whether there are any differences in the ability of subjects to activate the TrA during these exercises. Therefore, the purpose of this study was to measure TrA muscle thickness changes using the activation ratio during ADIM and quadruped exercises between healthy and non-symptomatic LBP (nLBP) patients. A secondary purpose was to examine TrA muscle thickness change within each group during the exercises.

## 2. Methods

A single-blinded case-control study was used. RUSI measurements were made by an assessor who was unaware of group membership (healthy control or LBP). The study was approved by the Investigational Review Board of Health Sciences Research (HSR no. 14092).

### 2.1. Subjects

Sixty subjects, 30 participants with nLBP and 30 healthy controls, from a local university community volunteered to participate in the study. The demographics for the groups are shown in Table 1. There were no differences between the groups during testing. Participants in the healthy group met the inclusion criteria of never having LBP. The inclusion criteria for the nLBP group were 3 or more episodes of LBP in the past year that resulted in limited activities of daily living or 5 or more episodes in their lifetime [21]. The nLBP group had to have an Oswestry Disability Index (ODI) score of 20% or worse. The subjects were asked to fill this out as if they were experiencing an episode of LBP, since we were recruiting people not currently having an episode of LBP. 

 Participants were excluded if they had a history of surgery in the lumbar spine, radiculopathy, were currently pregnant, had a chronic systemic or connective tissue disease, or had a balance disorder. Participants who believed that they met the advertised inclusion criteria for the nLBP group were prescreened over the phone to discuss inclusion/exclusion criteria prior to signing the consent form. All subjects read and signed written informed consent to participate in this study.

### 2.2. Instruments

#### 2.2.1. Ultrasound Imaging

Ultrasound imaging was obtained using a portable ultrasound unit (Philips GE Logiq Book XP, Milwaukee, WI) with a 7 MHz linear array probe that produced a 25 × 7 mm footprint. The intrarater ICC for B-mode ultrasound has previously been found to be .989 [22], and pilot testing in this study had similar reliability of .935 (CI =  .840–.977). The pilot testing consisted of measuring the TrA during the ADIM and quadruped exercises in 6 healthy people prior to data collection. Both MRI and EMG have validated RUSI when morphology and TrA activation were assessed [13, 23–28], however, not during the quadruped exercise.

### 2.3. Testing Procedures

Participants were given an overview of the abdominal musculature and the exercises to be performed. The subjects were randomly assigned to start with either the ADIM exercise or quadruped exercise. The resting images of the TrA were always obtained prior to the contracted images. Only the right side was tested when measuring the TrA, as described by Teyhen et al. [15]. For each exercise, the subjects were allowed 3 practice trials with clinician feedback on proper performance of all the exercises before imaging was collected as described by Springer et al. [29].

#### 2.3.1. Abdominal Drawing-In Maneuver

**Figure 1:**
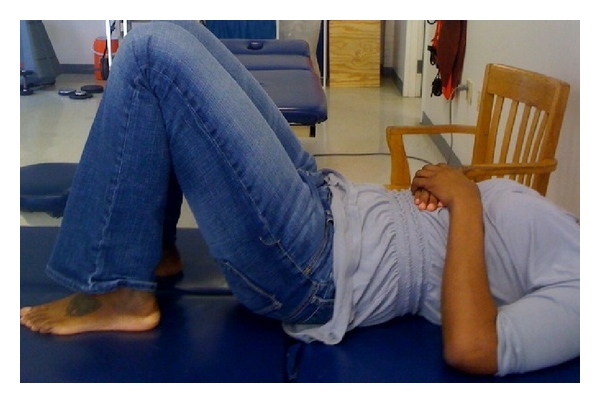
Rest position for ADIM.

The subjects were instructed to lie supine with knees bent to 90 degrees (Figure 1). This was the starting or rest position. To perform the contraction, the patient was instructed to “take a breath in and after you exhale pull your belly button in and back towards your spine” [15]. 

#### 2.3.2. Quadruped Exercise

**Figure 2:**
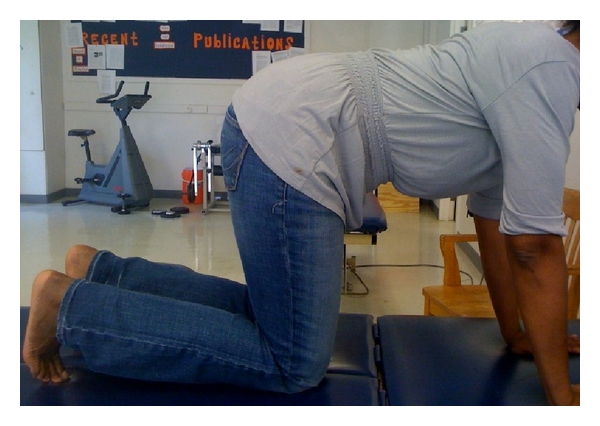
Starting position for the quadruped exercise.

**Figure 3:**
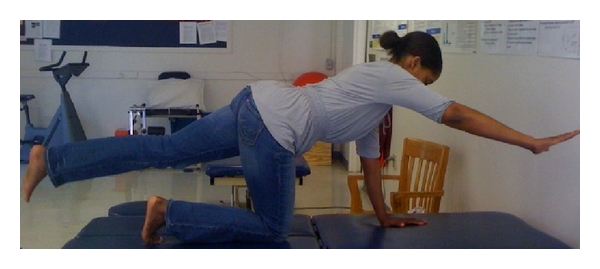
End positioning for the quadruped exercise.

As described by Teyhen et al. [15], the participants started (resting position) on their hands and knees, with a flat back, while looking straight ahead (Figure 2). To contract the TrA in this position, the participant was instructed to perform the ADIM and then raise the right arm and left leg simultaneously until the extremities were level with the trunk (Figure 3). This position was held for five seconds while the right side of the TrA was imaged and then the patient returned to the starting position. During the five seconds hold, the patient was told not to rotate or sag the trunk. After resting in the starting position for 30 seconds, the patient performed the exercise again.

#### 2.3.3. Ultrasound Imaging Procedure

The transducer was placed just superior to the iliac crest on the right side in the transverse plane along the midaxillary line [30]. To standardize the placement of the transducer among subjects, the hyperechoic interface between the TrA and the thoracolumbar fascia was the right most structure of the ultrasound image. The angle of the transducer was altered to ensure that the best image was captured and the fascial layers of the abdominal muscles were parallel on the screen [30]. Three images were recorded in the rest position and three images were recorded in the contracted position for both exercises. The image was recorded during the end of exhalation to standardize when images were recorded across participants, as well as to limit the effect of respiration of muscle thickness [30]. A second clinician who was blinded to the condition of the subjects measured all images at a later time from the top of the superficial TrA fascial layer to the beginning of the inferior fascial layer of the TrA. The average of the three measurements in each state was used in the statistical analysis.

#### 2.3.4. Statistical Analysis

**Figure 4:**
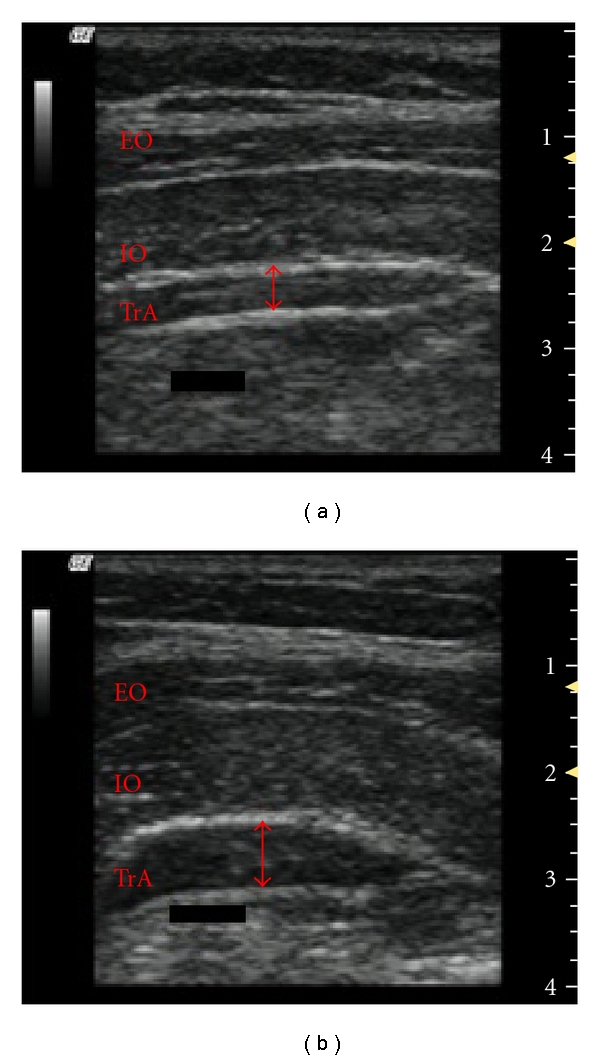
Ultrasound image of the TrA and calculation of TrA activation ratio. Resting image is on (a) and contracted image is on (b) (Activation ratio = TrA contracted (mm)/TrA rest (mm)). EO = external oblique; IO = internal oblique; TrA = transverse abdominis. The red arrow indicates the thickness of the TrA.

The independent variables were subject condition (healthy or nLBP), exercise type (ADIM and quadruped), and exercise state (rest and contraction). The dependent variable was TrA activation ratio between healthy and LBP patients. The TrA activation ratio was determined by dividing the contracted muscle thickness by the relaxed muscle thickness (Figure 4) as previously described [30]. For example, a ratio of 2 indicates a muscle thickness change 2 times greater than that of the resting state. Paired *t*-tests were used to compare (1) resting thickness between groups, (2) thickness from rest to contraction, (3) activation ratios between ADIM and quadruped in each group, and (4) activation ratios between the nLBP and healthy groups during each exercise. A power analysis was conducted using data from the study by Teyhen et al. in [15].

## 3. Results

At rest prior to the ADIM, there was no difference in TrA thickness (*P* = .919) between those with nLBP and the healthy group. There was also no difference in TrA thickness (*P* = .517)  at rest for the quadruped exercise between those with nLBP and the healthy group. The activation ratios between healthy and nLBP groups were not different from each other during the ADIM exercise (*P* = .254) nor the quadruped exercise (*P* = .463). There were, however, significant differences between the ADIM and quadruped activation ratios among the healthy (*P* < .001) and nLBP (*P* < .001) groups. TrA activation ratio means and standard deviations are presented in Table 2.

## 4. Discussion

Both the healthy and nLBP groups were able to contract the TrA during the two exercises, as indicated by an activation ratio greater than 1, with no significant differences between the two groups. However, there were differences in the TrA activation ratios between exercises, implying that the ADIM exercise did a better job at contracting the TrA. The ability of the exercises to activate the TrA is consistent with other researchers' findings in healthy individuals [15, 19, 30], but to our knowledge, this is the first study to compare the activation of the TrA between healthy and nLBP subjects using RUSI in both the ADIM and quadruped exercises. The lack of a difference between the healthy and nLBP groups may suggest that verbal instruction and feedback from a clinician may be enough to teach a person how to perform the exercises correctly. However, LBP still has a high recurrence rate, even after rehabilitation. The timing of when the TrA is activated prior to movement and how to train the TrA to become activated at the correct time are unclear. With delayed activation, but proper contraction of the TrA, the spine may be supported in a position of malalignment, resulting in LBP over time. 

The activation ratios in our study were lower than those reported by others. Previous studies report an activation ratio of 1.75 for healthy subjects during the ADIM [15], 1.6 for healthy subjects during the quadruped exercise [15], and 2.3 for LBP subjects during an ADIM [30]. For our study, the activation ratios were lower: with 1.52 for healthy subjects during the ADIM, 1.43 for healthy subjects during the quadruped exercise, 1.60 for nLBP subjects during the ADIM, and 1.49 for nLBP subjects during the quadruped. Our study population included individuals with variable activity levels, while others have investigated military recruits with higher activity levels. We may have seen lower activation ratios due to smaller muscle thickness changes. Activation seems to be dependent on the task, as well as how forceful the contraction is [21]. Although contraction force was not measured, the subjects performed the ADIM and quadruped exercises at a low force, which has been shown to correlate well to EMG recordings of TrA activation [20, 25]. We also used two 5-minute training periods to have the subjects perform the ADIM and quadruped correctly. This is a shorter time period that has been previously reported to be of 15 minutes. Although our results were different from those of previous studies, the activation of the TrA during the ADIM and quadruped was similar between groups during the task, indicating that the TrA may be involved in completing the exercise successfully. The TrA may also not be at rest when in the quadruped position resulting in less of an increase in TrA muscle thickness, resulting in a lower activation ratio. This exercise is also more complex, resulting in more constraints on the system. Therefore, there is less variability in the way the subject can perform the exercise [31, 32]. It is possible that the TrA is not as involved in performing the quadruped as in the ADIM, so smaller activation was noticed in the quadruped exercise. The function of the TrA in the nLBP group may also indicate that the normal function of the stabilizing muscles may return when pain subsides, contrary to a previous study [13]. 

The amount of training provided prior to RUSI was consistently performed using a prescripted format. The participants were given verbal and tactile feedback while the clinician viewed the US image; however the participants could not visualize the screen during training. Once a consistent performance of the ADIM and quadruped exercises was achieved, the testing began. Prior knowledge of the exercise, previous rehabilitation programs, or participation in core stability prevention programs may affect the TrA activation ratio. More research is needed for normative data on the TrA ratio. There has been shown to be a neuromuscular learning curve associated with learning new exercises [15], but this was not seen in our study.

The similarity in the activation of the TrA in each group implies that the ADIM and the quadruped exercises may be learned if using verbal cueing during a prevention or rehabilitation program. RUSI is not available in every clinical setting, but verbal instruction and practice seems to be enough to allow the person to contract the TrA. This is important for clinicians who do not have access to RUSI, but the activation of the TrA is warranted. Patients who are correctly classified into the stabilization group using clinical predictor rules may benefit from both of these exercises, since they are best treated with therapeutic exercise [7, 8]. Our nLBP subjects were not currently experiencing an episode of LBP and may not have fit into the stabilization category if clinical predictor rules would have been used for inclusion into the study. Teyhen et al. [33] used a subgroup of people experiencing LBP and found differences within a control group experiencing no pain. Our results may have changed if our inclusion criteria were more stringent and the nLBP subjects were currently in an episode of LBP. Similar to other exercise programs, continued practice and voluntary activation of a targeted muscle may improve the neuromuscular pathway to the muscle. If the voluntary neuromuscular activation of the muscle is exercised, the TrA may become more synchronized with extremity movements, resulting in more efficient stabilization. 

Previous researchers have associated a delayed activation of the TrA with nonspecific LBP [34, 35]. Our results indicate that these individuals are able to recruit and contract the TrA similar to healthy controls, but we were not able to measure the timing of muscle contractions with RUSI. Furthermore, other deep, stabilizing muscles should be examined such as the multifidus that has been reported to be atrophied in patients with LBP [9, 10, 19].

## 5. Conclusions

Both the ADIM and quadruped exercises may be appropriate in targeting the TrA during a rehabilitation or preventative program in people with LBP since these exercises activate the TrA. A training program to contract the TrA prior to performing exercise may benefit people with LBP since exercises that target the TrA have been shown to decrease pain and increase stability of the spine. With further training and possible progression of the ADIM and quadruped exercises to become more functional, these exercises could be used to target the TrA since it is involved in completing these exercises.

## Figures and Tables

**Table 1:** Descriptive statistics. Values represent means (SD).

Description	Healthy Group (*n* = 30)	nLBP Group (*n* = 30)
Age (y)	21.41 (.56)	24.53 (1.61)
Height (cm)	174.65 (1.82)	175.70 (2.04)
Weight (kg)	74.45 (2.71)	79.44 (3.50)
Oswestry Disability Index (%)	N/A	32.93 (2.36)

**Table 2:** Activation ratios comparing nLBP to healthy participants.

	Healthy	nLBP	*P*-value between groups
ADIM	1.52 ± .266	1.60 ± .316	.254
Quadruped	1.43 ± .257	1.49 ± .376	.463
*P*-value within groups	<.001	<.001	
